# ﻿Morphological, ecological, and molecular phylogenetic approaches reveal species boundaries and evolutionary history of *Goodyeracrassifolia* (Orchidaceae, Orchidoideae) and its closely related taxa

**DOI:** 10.3897/phytokeys.212.91536

**Published:** 2022-11-04

**Authors:** Kenji Suetsugu, Shun K. Hirota, Narumi Nakato, Yoshihisa Suyama, Shunsuke Serizawa

**Affiliations:** 1 Department of Biology, Graduate School of Science, Kobe University, Kobe 657-8501, Sakyo, Japan Kobe University Sakyo Japan; 2 Field Science Center, Graduate School of Agricultural Science, Tohoku University, 232-3 Yomogida, Naruko-onsen, Osaki, Miyagi, 989-6711, Japan Tohoku University Osaki Japan; 3 Narahashi 1–363, Higashiyamato, Tokyo 207-0031, Japan Unaffiliated Higashiyamato Japan; 4 Aichi Green Association, Urahata 198-1, Nagamaki, Oharu-sho, Aichi 490-1131, Japan Aichi Green Association Aichi Japan

**Keywords:** chromosome, cryptic species, integrative taxonomy, MIG-seq, phylogeny, reproductive biology, species complex

## Abstract

Species delimitation within the genus *Goodyera* is challenging among closely related species, because of phenotypic plasticity, ecological variation, and hybridization that confound identification methods based solely on morphology. In this study, we investigated the identity of *Goodyeracrassifolia* H.-J.Suh, S.-W.Seo, S.-H.Oh & T.Yukawa, morphologically similar to *Goodyeraschlechtendaliana* Rchb.f. This recently described taxon has long been known in Japan as “Oh-miyama-uzura” or “Gakunan” and considered a natural hybrid of *G.schlechtendaliana* and *G.similis* Blume (= *G.velutina* Maxim. ex Regel). Because the natural hybrid between *G.schlechtendaliana* and *G.similis* was described as *G.×tamnaensis* N.S.Lee, K.S.Lee, S.H.Yeau & C.S.Lee before the description of *G.crassifolia*, the latter might be a synonym of *G.×tamnaensis*. Consequently, we investigated species boundaries and evolutionary history of *G.crassifolia* and its closely related taxa based on multifaceted evidence. Consequently, morphological examination enabled us to distinguish *G.crassifolia* from other closely related species owing to the following characteristics: coriaceous leaf texture, laxly flowered inflorescence, long pedicellate ovary, large and weakly opened flowers, and column with lateral appendages. Ecological investigation indicates that *G.crassifolia* (2*n* = 60) is agamospermous, requiring neither pollinators nor autonomous self-pollination for fruit set, whereas *G.schlechtendaliana* (2*n* = 30) is neither autogamous nor agamospermous but is obligately pollinator-dependent. MIG-seq-based phylogenetic analysis provided no evidence of recent hybridization between *G.crassifolia* and its close congeners. Thus, molecular phylogeny reconstructed from MIG-seq data together with morphological, cytological, and ecological analyses support the separation of *G.crassifolia* as an independent species.

## ﻿Introduction

The genus *Goodyera* R.Br. (Orchidaceae, Orchidoideae, Cranichideae) includes ca. 70 species distributed in Africa, Europe, the Western Indian Ocean Islands, Asia, the southwestern Pacific Islands, northeastern Australia, North America, and Mesoamerica (Govaerts et al. 2022). *Goodyera* spp. are terrestrial, lithophytic or epiphytic, and typically grow under shade, on mossy rocks, or along moist tracks of perennial mountain streams (Pridgeon et al. 2003). The characteristic features of the genus include creeping rhizomes; evergreen foliage that often features white or golden venation on the upper surface; and flowers with saccate lips, a single stigmatic lobe, and two sectile pollinia attached to a viscidium (Pridgeon et al. 2003). The flowers present dissimilar sepals and a concave dorsal sepal that forms a hood over the column along with the petals. The lateral sepals are usually connivent, with a lip that is formed from the concave-saccate hypochile and sessile epichile (Guan et al. 2014; Suetsugu and Hayakawa 2019).

The identification of species within *Goodyera* is often a challenge, especially among closely related species, owing to attributes such as phenotypic plasticity, convergent morphological features, and hybridization (Kallunki 1976, 1981; Hu et al. 2016; Suetsugu et al. 2019, 2021a); these eventually hinder tracing the evolutionary history of the genus (Pace 2020). Notably, molecular techniques have recently emerged as invaluable tools for investigating phylogenetic relationships within *Goodyera* (Hu et al. 2016; Suetsugu et al. 2021a). In particular, the internal transcribed spacer (ITS) region of nuclear ribosomal DNA—which exhibits moderate interspecific variation—has served as a primary target for phylogenetic analysis to determine the lower taxonomic levels of plants (Baldwin et al. 1995; Guan et al. 2014). In *Goodyera*, however, the ITS sequences of the morphologically distinct species *G.similis* Blume (= *G.velutina* Maxim. ex Regel) and *G.repens* (L.) R.Br. are identical (Shin et al. 2002). Therefore, phylogenetic resolution may be insufficient for species identification in *Goodyera*. Furthermore, the findings of a more comprehensive phylogenetic study including data from ITS and plastid regions (*trnL-F* and *matK*) could not be correlated with the corresponding species identification using morphological characteristics (Hu et al. 2016). Therefore, a higher resolution genetic marker is needed to elucidate the complex evolutionary history of *Goodyera* species (Suetsugu et al. 2021a, b).

A potential solution to distinguish closely related species would be to implement a high-throughput sequencing technology that enables simultaneous sequencing of numerous loci (Suyama and Matsuki 2015). Indeed, high-throughput sequencing has helped determine the boundaries and evolutionary histories of closely related species (Tamaki et al. 2017; Yoichi et al. 2018; Hirano et al. 2019; Suetsugu et al. 2021a). For example, multiplexed inter-simple sequence repeat (ISSR) genotyping by sequencing (MIG-seq) has recently been identified as a powerful tool for detecting reproductive isolation and hybridization, even between recently diverged species, including closely related *Goodyera* species (Tamaki et al. 2017; Yoichi et al. 2018; Hirano et al. 2019; Suetsugu et al. 2021a).

Ecological data based on breeding systems can further clarify whether morphologically distinct populations should be considered separate, reproductively isolated species (Kallunki 1981; Coyne and Orr 2004; Botes et al. 2020). In the present study, we investigated the identity of *Goodyeracrassifolia* H.-J.Suh, S.-W.Seo, S.-H.Oh & T.Yukawa—recently described in Korea and Japan (Oh et al. 2022)—using a multifaceted approach. *Goodyeracrassifolia* is morphologically the most similar to *G.schlechtendaliana* Rchb.f. and often grows sympatrically with the latter. *Goodyeracrassifolia* has long been recognized as “Oh-miyama-uzura (meaning larger *G.schlechtendaliana*)” or “Gakunan (named after the collection site)” in Japan, differing from *G.schlechtendaliana* by its larger stature, more coriaceous leaves with indistinct reticulation, and more laxly flowered inflorescences (Takahashi 1985; Serizawa 2008; Akiyama 2010). Although the taxon had not been formally described until recently, it was often considered a natural hybrid of *G.schlechtendaliana* and *G.similis* (Takahashi 1985; Akiyama 2010; The Flora-Kanagawa Association 2018). Notably, the natural hybrid between *G.schlechtendaliana* and *G.similis* was described as *G.×tamnaensis* in Jeju Island, South Korea (Lee et al. 2010, 2012). Suetsugu et al. (2021b) later reported the first occurrence of *G.×tamnaensis* on the Boso Peninsula, Chiba Prefecture, Japan. Given that *G.×tamnaensis* was described before *G.crassifolia*, it is possible that *G.crassifolia* is a junior synonym of *G.×tamnaensis.* However, the report by Oh et al. (2022) did not include a comparison between *G.crassifolia* and *G.×tamnaensis*.

In this study, we used an integrative taxonomic approach to investigate species boundaries and evolutionary history of *G.crassifolia* and its closely related taxa. Species delimitation that explicitly considers ecological as well as phylogenetic differences represents a crucial step in our understanding of biodiversity (Barrett and Freudenstein 2011). Over the last two decades, integrative taxonomy has helped achieve more robust estimates of biodiversity than those based on one-dimensional representations of variation (such as morphology), especially in the case of taxonomically challenging species (Barrett and Freudenstein 2011; Botes et al. 2020; Barrett et al. 2022). Our multifaceted evidence leads us to conclude that *G.crassifolia* is morphologically, phylogenetically, and ecologically distinct from *G.schlechtendaliana* and *G.×tamnaensis* and should, therefore, be considered as a separate species.

## ﻿Materials and methods

### ﻿Morphological observations

We compared the morphological characters of *G.crassifolia*, *G.schlechtendaliana*, *G.×tamnaensis*, and *G.similis* from herbarium specimens deposited in AICH, HIBG, HYO, KYO, MAK, SCM, TI, and TNS and from living plants collected throughout Japan during fieldwork between 2011 and 2021. Morphological variations among *G.schlechtendaliana*, *G.×tamnaensis*, and *G.similis* were further investigated by reviewing the literature. Morphological characters were visually observed under a Leica M165C stereomicroscope and measured using a digital caliper. The dissected floral parts were photographed using an Olympus OM-D E-M1 Mark II digital camera equipped with an Olympus 30 mm macro lens or a Leica MC170 HD digital camera attached to a Leica M165C stereo microscope. Since we revealed that *G.crassifolia* is distributed widely throughout Japan, we also provided a revised description of *G.crassifolia* based on the newly discovered specimens from our field surveys and herbarium investigations. At least one voucher specimen from each new population discovered during our field survey was deposited in KYO and TNS (Suppl. material 1). The herbarium acronyms follow Index Herbariorum (Thiers 2022).

### ﻿Cytological observations

Root tips were collected from five individuals of *G.crassifolia* (representing five populations) and four individuals of *G.schlechtendaliana* (including a G.schlechtendalianavar.yakushimensis Suetsugu & H.Hayak. individual; representing three populations). They were used for mitotic chromosome counts, as described in Suetsugu et al. (2019). Root tips were pretreated with 2 mM 8-hydroxyquinoline solution for 4–5 h, fixed in Carnoy’s solution for 1–24 h, macerated in 1 N HCl at 60 °C for 1 min, and then squashed in aceto-orcein. The samples were then observed and photographed under a light microscope.

### ﻿Breeding system

The breeding systems of *G.schlechtendaliana* and *G.crassifolia* were investigated during early-to-late September 2016 in a sympatric population in Kami-shi, Kochi Pref., Japan. Hand-pollination experiments were performed using five treatments: (i) agamospermous treatment—the pollinaria were removed before anthesis using forceps, and the flowers were then bagged (20 flowers from five individuals); (ii) autonomous autogamous treatment—flowers were bagged with a fine-meshed net before anthesis to exclude pollinators (20 flowers from five individuals); (iii) manually autogamous treatment—the pollinaria were removed and used to hand-pollinate the same flower before bagging (20 flowers from five individuals); (iv) manually allogamous treatment—same as treatment (iii) but using the pollinia from a different plant at least 1 m from the recipient plant (20 flowers from five individuals); and (v) open treatment—flowering individuals were randomly tagged and allowed to develop fruit under natural conditions (40 flowers from 10 individuals). The experimental plants were monitored intermittently over the subsequent 4–6 weeks; fruit set among the treatments was compared via Fisher’s exact test. Mature fruits were collected and silica-dried; seed mass was obtained to the nearest 0.0001 g. Thereafter, 200 seeds per capsule were examined to assess the presence of the embryo. After confirming the normality and homogeneity of variance using the Shapiro-Wilk and Bartlett’s tests, the effects of pollination treatment on the seed mass and the proportion of seeds with at least one embryo were tested via ANOVA.

### ﻿MIG-seq-based high-throughput genomic analysis

Eleven *G.crassifolia* individuals representing six populations, ten *G.schlechtendaliana* individuals (including five of G.schlechtendalianavar.yakushimensis), and fifteen *G.similis* individuals were collected throughout Japan. Three individuals of *G.×tamnaensis*, a natural hybrid between *G.schlechtendaliana* and *G.similis* (Lee et al. 2010, 2012; Suetsugu et al. 2021b), were included in the comparative study (Suppl. material 1). Genomic DNA was extracted from silica-dried leaves using the CTAB method. An MIG-seq library for the 39 *Goodyera* samples was prepared according to the protocol outlined in Suyama et al. (2022). The library was sequenced using an Illumina MiSeq Sequencer (Illumina, San Diego, CA, USA) with a MiSeq Reagent Kit v3 (150 cycle, Illumina). The raw MIG-seq data of the 15 *G.similis* samples, 10 *G.schlechtendaliana* samples (including five G.schlechtendalianavar.yakushimensis samples), and three *G.×tamnaensis* samples had previously been deposited at the DDBJ Sequence Read Archive (DRA, accession number DRA011506) for Suetsugu et al. (2021b). The raw MIG-seq data of the 11 *G.crassifolia* samples were deposited at the DDBJ Sequence Read Archive (DRA, accession number DRA014540).

After removing the primer sequences and low-quality sequencing reads (Suetsugu et al. 2021b), 3 594 716 reads (92 172 ± 3937 reads per sample) were obtained from 4 058 158 raw reads (104 055 ± 4344 per sample). Stacks 2.60 pipeline was used for *de novo* single nucleotide polymorphism (SNP) discovery (Rochette et al. 2019), with the following parameters: minimum depth of coverage required to create a stack (*m*) = 3, maximum distance allowed between stacks (*M*) = 2, and number of mismatches allowed between sample loci while building the catalog (*n*) = 2. For the maximum likelihood and SplitsTree phylogenetic analyses, SNPs retained by four or more samples were used; for the population structure analysis, SNPs retained by 16 or more samples were used. SNPs with high heterozygosity (Ho ≥ 0.6) were removed. SNP sites with fewer than three minor alleles were filtered out. Finally, 4790 SNPs from 2795 loci were retained for phylogenetic analysis. For STRUCTURE analysis, to avoid linked SNPs, we used only the first SNP from each locus, retaining 874 SNPs.

Our SNP-based maximum likelihood phylogeny was inferred using RAxML 8.2.10 (Stamatakis 2014), using a GTR substitution model with Lewis’ ascertainment bias correction and 1000 iterations of parallelized tree search bootstrapping. To examine interspecific hybridization, a Neighbor-Net network was constructed using SplitsTree4 4.14 (Huson and Bryant 2006) using the uncorrelated P distance matrix. Population structure was examined using STRUCTURE 2.3.4 (Pritchard et al. 2000). We performed 20 independent runs, with a burn-in of 100 000 steps and an additional 100 000 steps using an admixture model, and estimated the log-likelihoods for each cluster (*K* = 1–10). Optimal *K* values were determined using the Delta K method (Evanno et al. 2005) in Structure Harvester (Earl and vonHoldt 2012). The results were visualized using CLUMPAK (Cluster Markov Packager Across K) (Kopelman et al. 2015).

## ﻿Results and discussion

### ﻿Morphological distinctness of *Goodyeracrassifolia*

**Figure 1.**
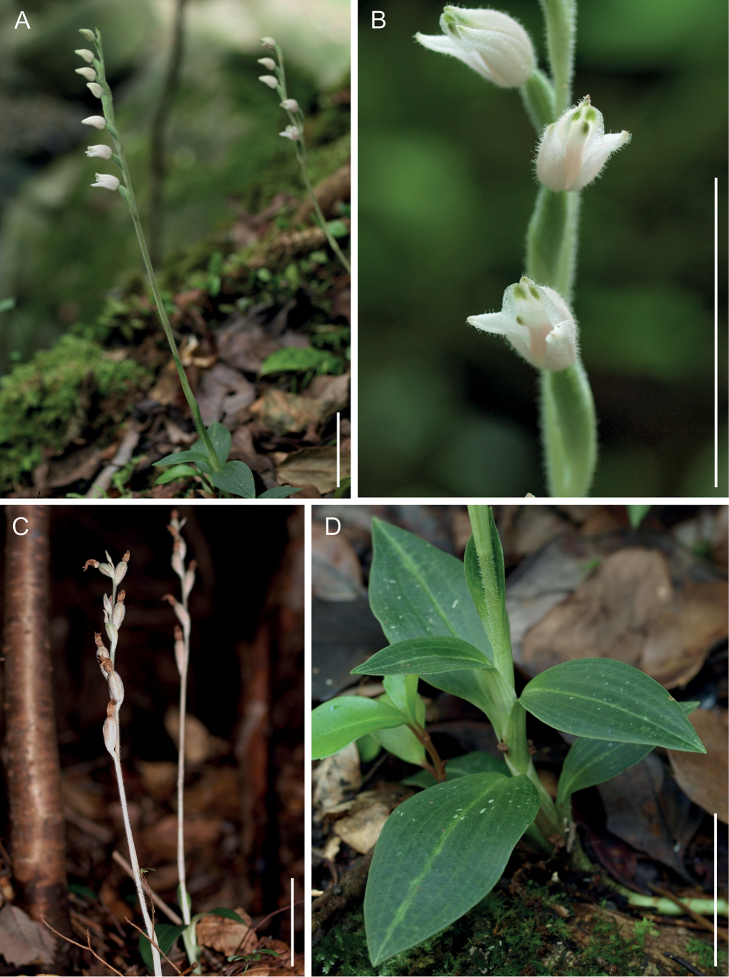
*Goodyeracrassifolia* in its natural habitat **A** flowering individual **B** flowers **C** fruiting individual **D** leaves. Scale bars: 30 mm.

**Figure 2.**
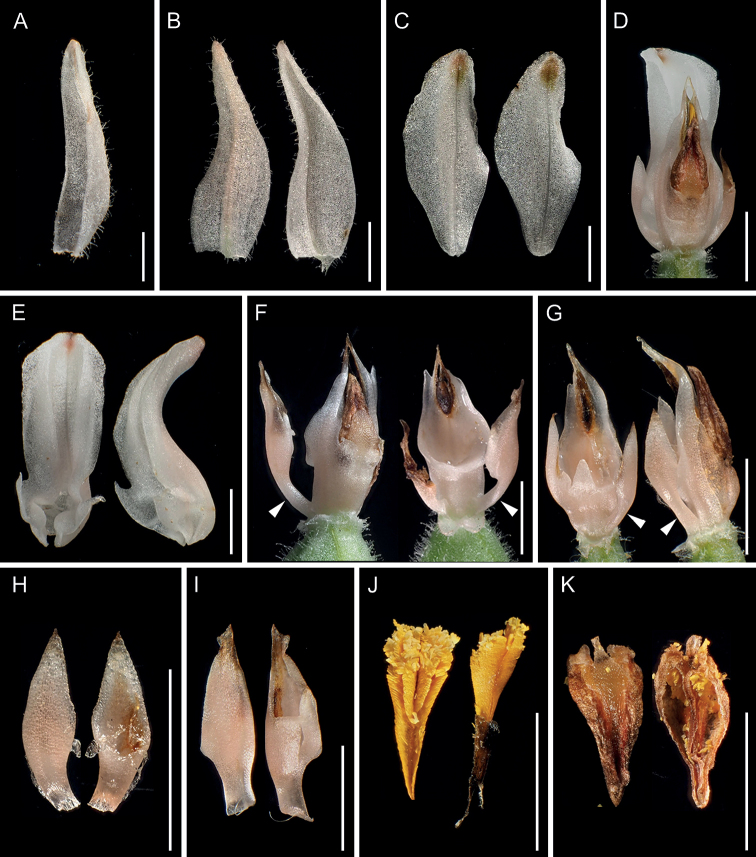
*Goodyeracrassifolia* from Kami City, Kochi Prefecture (*Hisanori Takeuchi G161-1*, KYO) **A** dorsal sepal (abaxial view) **B** lateral sepals (left: abaxial view, right: adaxial view) **C** lateral petals (left: abaxial view, right: adaxial view) **D** lip and column (dorsal view) **E** lip (left: adaxial view, right: lateral view) **F** column (left: obliquely dorsal view, right: ventral view) **G** column (left: ventral view, right: lateral view) **H** lateral appendages removed from column (left: dorsal view, right: ventral view) **I** lateral appendages removed from column (both: dorsal view) **J** pollinarium (left: dorsal view, right: ventral view) **K** anther cap (left: dorsal view, right: ventral view). Arrows indicate the conspicuous lateral appendages. Photographs except **G** and **I** are derived from the same flower. **G** and **I** are used to show morphological variation of column within the same individual. Scale bars: 3 mm.

**Figure 3.**
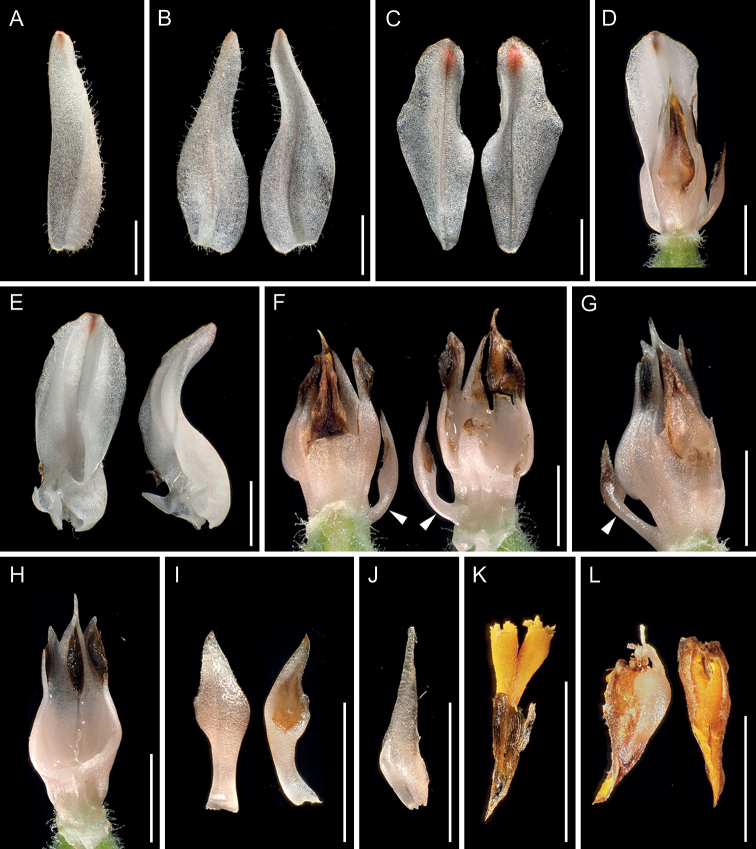
*Goodyeracrassifolia* from Higashimuro County, Wakayama Prefecture (*Yasuo Takada s.n.*, KYO) **F, G** column The conspicuous lateral appendages are indicated by arrows **H** column removing lateral appendages **I, J** lateral appendages removed from column **K** pollinarium **L** anther cap and pollinarium **A** dorsal sepal (abaxial view) **B** lateral sepals (left: abaxial view, right: adaxial view) **C** lateral petals (left: abaxial view, right: adaxial view) **D** lip and column (dorsal view) **E** lip (left: adaxial view, right: lateral view) **F** column (left: dorsal view, right: ventral view) **G** column (obliquely lateral view) **H** column removing lateral appendages (ventral view) **I** lateral appendages removed from column (left: dorsal view, right: ventral view **J** lateral appendages removed from column (ventral view) **K** pollinarium (ventral view) **L** anther cap and pollinarium (left: dorsal view, right: ventral view). Arrows indicate the conspicuous lateral appendages. Photographs except **G, H, J, K** are derived from the same flower **G, H, J** show the variation of column morphology within the same individual, while **K** is used because pollinaria were detached from anther cap of a flower that was mainly used. Scale bars: 3 mm.

**Figure 4.**
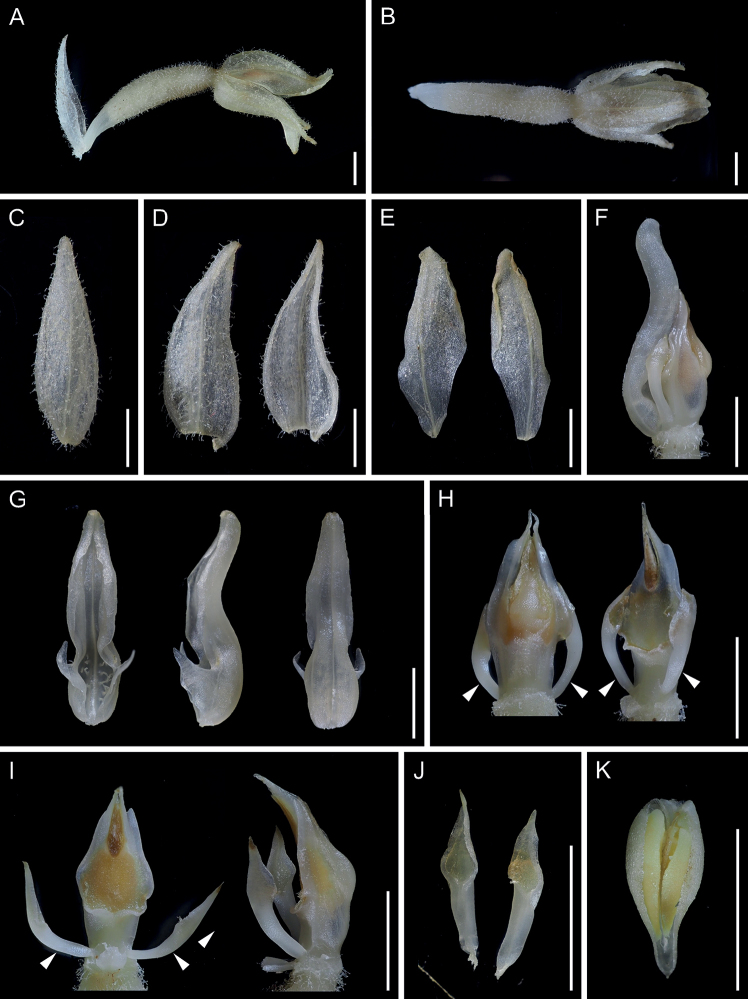
*Goodyeracrassifolia* (*Koji Tanaka KS209*, KYO; photographed after immersion in 50 percent ethanol) **A** flower (lateral view) **B** flower (dorsal view) **C** dorsal sepal (abaxial view) **D** lateral sepals (left: abaxial view, right: adaxial view) **E** lateral petals (left: abaxial view, right: adaxial view) **F** lip and column (lateral view) **G** lip (left: adaxial view, middle: lateral view, right: abaxial view) **H** column (left: dorsal view, right: obliquely ventral view) **I** column with partially detached lateral appendages (left: ventral view, right: lateral view) **J** lateral appendages removed from column (ventral view) **K** anther cap and pollinarium (ventral view). Arrows indicate the conspicuous lateral appendages. All photographs are derived from the same flower. Scale bars: 3 mm.

**Figure 5.**
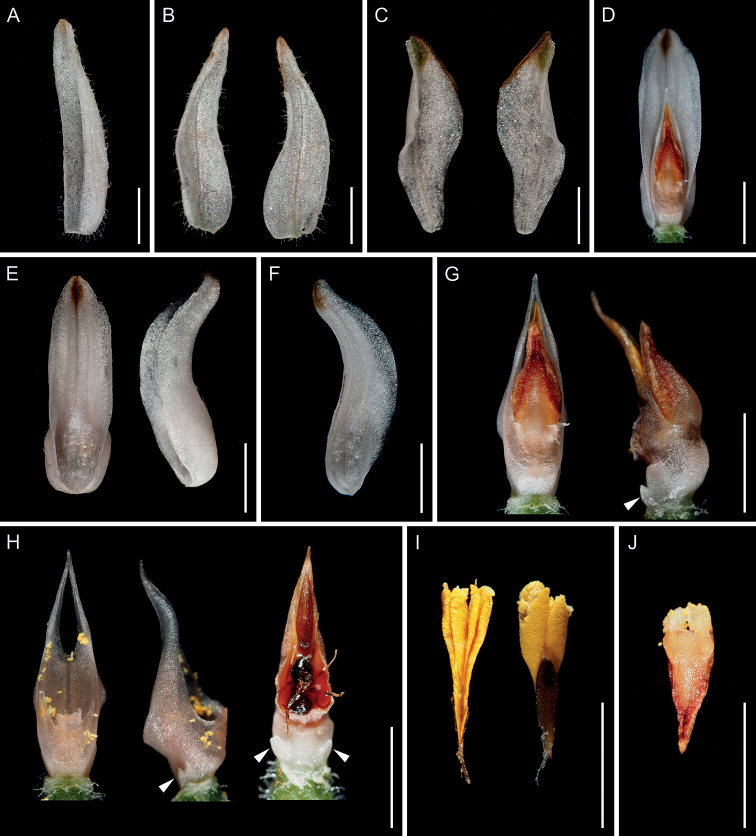
*Goodyeracrassifolia* (*Hisanori Takeuchi & Kenji Suetsugu KS208*, KYO) **A** dorsal sepal (adaxial view) **B** lateral sepal (adaxial view) **C** lateral petal (adaxial view) **D** lip and column (dorsal view) **E** lip (left: adaxial view, right: lateral view) **F** longitudinal section of lip (adaxial view) **G** column and anther (left: top view, right: lateral view) **H** column (left: dorsal view, middle: lateral view, right: ventral view) **I** pollinarium (left: dorsal view, right: ventral view) **J** anther cap (dorsal view). Arrows indicate the conspicuous lateral appendages. All photographs are derived from the same flower. Scale bars: 3 mm.

The most remarkable characteristic of *G.crassifolia* is its column with lateral appendages (Figs 1–5). The lateral column appendages are consistently absent in the closely related taxa. Since the lateral appendages are themselves column-like, they are likely to be enlarged staminodes (Oh et al. 2022). Notably, the lateral appendages of the column differ significantly in size among populations, and in terms of their position on the inflorescence, being often conspicuous in the basal flowers and inconspicuous (or rarely absent) in the apical flowers. We observed an association between the column and lip or rostellum shape; the lip and the rostellum appeared to be three-lobed when the lateral appendages are conspicuous (Figs 2E, F, 3E, F, 4G, H, 5E, G). Given that the floral organ formation is explained mainly by the combined expression of ABCE-class MADS-box transcription factors (Causier et al. 2010; Hsu et al. 2015, 2021; Suetsugu et al. 2022), the spatial expression of the factors underlying this distinctive morphology deserves further investigation. In particular, the enlarged staminodes indicate that *G.crassifolia* exhibits some radial symmetry, unlike most orchid flowers, which are typically zygomorphic.

Detailed morphological examination revealed that *G.crassifolia* can be distinguished from *G.schlechtendaliana* by not only column shape (column with vs. without lateral appendages) but also plant height (20–37 cm vs. ca. 15 cm), leaf texture (coriaceous vs. papyraceous), leaf coloration (glossy green, with narrow pale-white reticulation, to green with no decorations vs. green with obvious and broad white reticulation), inflorescence architecture (lax, internodes 17–24 mm long at inflorescence base vs. dense internodes 6–10 mm long at inflorescence base), pedicellate ovary length (11–20 mm, longer than floral bract vs. 7–9 mm, as long as the floral bract), flower opening (opening weakly vs. widely), flower size (sepal and petal length > 10 mm vs. < 10 mm), shape of lateral sepal (recurved at two-thirds of its entire length from the base vs. strongly recurved at half its entire length from the base), hypochile shape (weakly vs. strongly concave-saccate), and seed shape (often polyembryonic vs. always monoembryonic) (Lee et al. 2010, 2012; Bhattacharjee and Chowdhery 2012; Suetsugu and Hayakawa 2019; Suetsugu et al. 2021b; Oh et al. 2022).

It should be noted that *G.crassifolia* has previously been confused with *G.×tamnaensis* in Japan (Takahashi 1985; Akiyama 2010; The Flora-Kanagawa Association 2018). In fact, *G.crassifolia* is superficially similar to *G.×tamnaensis* in terms of its weakly opening flowers but differs in plant height (20–37 cm for *G.crassifolia* vs. 10–15 cm for G.×tamnaensis), leaf texture (coriaceous vs. papyraceous), leaf coloration (glossy green with narrow, pale-white reticulation to green with no decoration on upper surface vs. velutinous dark green with a white central vein and reticulate venation), ovary and pedicel length (11–20 mm vs. 7–10 mm long), flower size (petal and sepal length > 10 mm vs. < 10 mm), column shape (column with vs. without lateral appendages), and rostellum shape (acuminate apex, occasionally bi- or trilobed vs. flattened and cuneate apex, never divided) (Lee et al. 2010, 2012; Bhattacharjee and Chowdhery 2012; Suetsugu and Hayakawa 2019; Suetsugu et al. 2021b).

Further detailed comparison of morphological characters among *G.crassifolia*, *G.schlechtendaliana* and *G.×tamnaensis* is given in Table 1. Additional descriptions and illustrations of *G.crassifolia*, *G.schlechtendaliana*, *G.×tamnaensis*, and *G.similis* are available in Lee et al. (2010, 2012), Suetsugu and Hayakawa (2019), Suetsugu et al. (2021b), and Oh et al. (2022).

**Table 1.** Morphological comparison among *Goodyeracrassifolia*, *G.schlechtendaliana*, G.×tamnaensis and *G.velutina*.

Characters	*G.crassifolia*	*G.schlechtendaliana*	*G.×tamnaensis*	*G.velutina*
inflorescence length	20–37 cm	ca. 15 cm	10–15 cm	6–10 cm
leaf texture	coriaceous	papyraceous	papyraceous	papyraceous
leaf color	glossy green	glossy green	velutinous dark green	velutinous dark green
leaf shape	ovate to lanceolate-ovate	elliptic-ovate	lanceolate-ovate	ovate
leaf central vein	faint	faint	prominent	prominent
leaf lateral vein	faint	prominent	intermediate	hidden
leaf reticulate venation	faint	prominent	faint	visually unrecognizable
ovary and pedicel length	11–20 mm	7–9 mm	7–10 mm	7–10 mm
hair shape and length on peduncle and ovary	0.3–0.5 mm, clavate	0.3–0.4 mm, clavate	0.3–0.4 mm, clavate	0.1 mm, subulate
color of bract, ovary and inflorescence	pale green	pale green	reddish-brown	reddish-brown
flower opening	weekly open	widely open	weekly open	weekly open
flower color	white	white	light reddish pink	light reddish pink
color of lip and lateral petal apex	usually dark brown or rarely brown	usually brown or rarely dark green	light reddish pink	light reddish pink
shape of lip apex	recurved	strongly recurved	recurved	slightly recurved
lateral column appendages	present or rarely absent	absent	absent	absent
rostellum shape	narrowly triangular, 1/2 as long as column, apex acuminate, occasionally bi- or trilobed	narrowly triangular, 1/2 as long as column, apex acuminate, never divided	narrowly triangular, 1/2 as long as column, apex cuneate, never divided	oblong to rectangular, 2/5 as long as column, apex cuneate, never divided

### ﻿Reproductive barriers between *Goodyeracrassifolia* and *G.schlechtendaliana*

Polyploidization is commonly accepted as a vital mechanism of sympatric speciation in plants (Köhler et al. 2010). Owing to chromosome number imbalance during meiosis, backcross between either parent would mostly result in nonviable progenies; those rare survivors with unbalanced chromosome numbers will be primarily sterile (Ramsey and Schemske 1998). The triploid-block is a significant reproductive barrier leading to polyploid speciation (Köhler et al. 2010).

**Figure 6.**
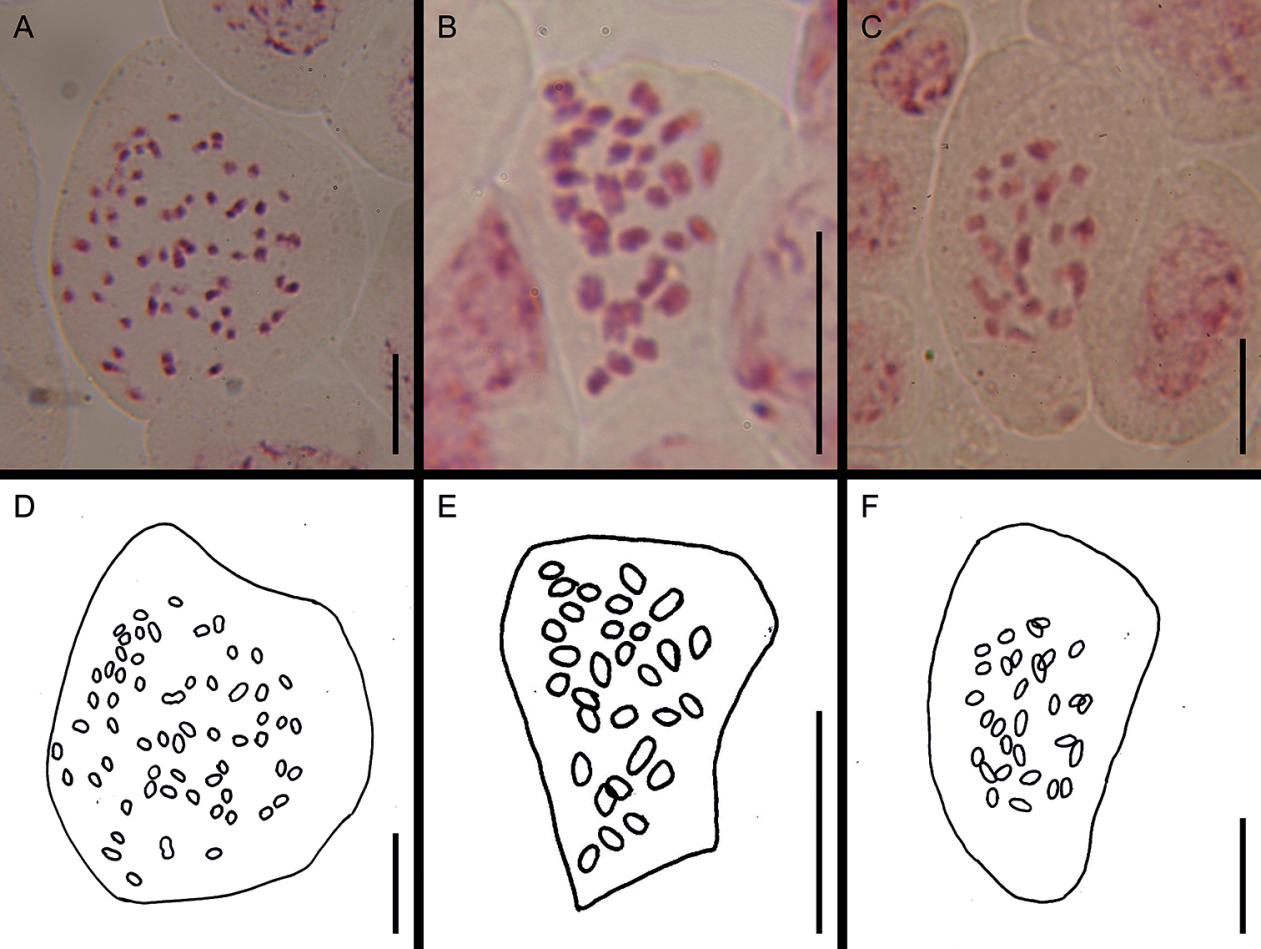
Somatic chromosomes (**A–C**) and their explanatory drawings (**D–F**) of *Goodyeracrassifolia* and its closely related taxa **A, D***G.crassifolia***B, E***G.schlechtendaliana***C, F**G.schlechtendalianavar.yakushimensis. Scale bars: 10 μm.

Investigation of chromosome numbers provided evidence of polyploidy in *G.crassifolia*: all of the *G.schlechtendaliana* individuals (including G.schlechtendalianavar.yakushimensis) showed a chromosome number of 2*n* = 30; whereas all *G.crassifolia* individuals (Fig. 6) showed 2*n* = 60. In line with the results obtained in this study, Oh et al. (2022) reported 2*n* = 60 for a Korean *G.crassifolia* individual. Intriguingly, Sera (1990) reported 2*n* = 60 in five “*G.schlechtendaliana*” plants from four localities, while reporting 2*n* = 30 for most *G.schlechtendaliana* individuals (60 plants from 22 localities collected throughout Japan). However, Sera (1990) noted that the 2*n* = 60 “*G.schlechtendaliana*” plants possess coriaceous leaves with faint reticulate variegation. The photographs listed in Sera (1990) indicate that they also have laxly flowered inflorescences and a longer pedicellate ovary, which are characteristic features of *G.crassifolia*. Although three of the voucher specimens from Sera (1990) have unfortunately been lost, possibly during the relocation of the herbarium HIBG (T. Sera, personal communication), we could identify the two remaining voucher specimens as *G.crassifolia*. It is likely that all of the 2*n* = 60 plants of Sera (1990) could be *G.crassifolia*. Given that 2*n* = 30 is the only chromosome number reported in *G.schlechtendaliana* as determined by other previous studies (Matsuura and Nakahira 1958; Shoji 1963; Tanaka 1965; Sun et al. 1996; Tae et al. 1997), 2*n* = 30 is arguably the typical chromosome number of *G.schlechtendaliana*. In addition, 2*n* = 30 has been reported in *G.×tamnaensis* (Lee et al. 2012), although speciation via hybridization without a change in chromosome number is considered rare (Schumer et al. 2014). Thus, as suggested by Oh et al. (2022), the cytological distinctness of *G.crassifolia* may have partially contributed to its reproductive isolation.

Our pollination experiments revealed the contrasting breeding systems of *G.crassifolia* and *G.schlechtendaliana*. The latter, although self-compatible, is neither autogamous nor agamospermous, and shows low fruit set under natural conditions; pollinator limitation was the major cause of low fruit set, which was significantly improved by manual autogamy and allogamy (Table 2, *P* < 0.001). By contrast, the natural populations of *G.crassifolia* consistently exhibited high fruit set (Fig. 1). Given that high fruit set was obtained in agamospermous, bagged, manually geitonogamous, manually allogamous, and open flowers, *G.crassifolia* flowers are not pollinator-limited under natural conditions. Neither seed mass nor the proportion of seeds with embryo varied significantly with pollination treatment (Table 2). Given that the rostellum functionally prevents autonomous autogamy, agamospermy is arguably the main cause of high fruit set in *G.crassifolia.* Therefore, agamospermy provides reproductive assurance under pollinator limitation in *G.crassifolia*.

**Table 2.** Effects of pollination treatment on fruit set, seed mass and proportion of seeds with embryo in *Goodyeracrassifolia* and *G.schlechtendaliana*.

Species		Agamospermy	Autonomous autogamy	Manual autogamy	Manual allogamy	Open
*G.crassifolia*	Fruit set (%)	85.0^a^	95.0^a^	90.0^a^	85.0^a^	87.5^a^
	Seed mass (mg)	8.1 ± 2.5^a^	8.1 ± 2.3^a^	7.9 ± 2.0^a^	7.9 ± 2.3^a^	8.1 ± 1.8^a^
	Seeds with embryo	165.7 ± 9.9^a^	163.4 ± 9.2^a^	164.2 ± 9.0^a^	164.1 ± 9.5^a^	162.6 ± 8.0^a^
*G.schlechtendaliana*	Fruit set (%)	0^a^	0^a^	90.0^b^	90.0^b^	32.5^c^
	Seed mass (mg)	–	–	2.8 ± 1.5^a^	3.4 ± 1.5^a^	3.1 ± 1.5^a^
	Seeds with embryo	–	–	185.8 ± 7.5^a^	187.1 ± 8.0^a^	187.2 ± 5.7^a^

Different superscript letters indicate significant differences (*P* < 0.05) between treatment groups. Both seed mass and seeds with embryo are expressed by mean ± SD.

Notably, the viscidium of *G.crassifolia* exhibits almost no adhesion, hindering its attachment onto its potential pollinators. No pollinia removal or deposition was observed during the field study. Because (i) *G.crassifolia* has weakly opened flowers with less-adhesive pollinia and (ii) its stigma is sometimes covered with column appendages (Figs 2G, 3F), there are arguably few opportunities for outcrossing. Thus, agamospermy is probably its dominant, if not exclusive, reproductive strategy. The reduced selection pressure on outcrossing may have led to the aforementioned variations in the lip, column appendages, and rostellum morphology, even within a single inflorescence. The polyembryony detected in *G.crassifolia* is further indicative of agamospermy, given that adventitious embryony, the most common form of apomixis, is characterized by a high number of polyembryonic seeds (Catling 1982; Campacci et al. 2017; Naumova 2018).

During our field study, we confirmed the phenological isolation between *G.crassifolia* and *G.schlechtendaliana* as previously reported by Takahashi (1985) and Oh et al. (2022). In many regions where both are sympatric (e.g., Hongdo, Korea: Oh et al. 2022; southern and central Japan: Takahashi (1985) and field observations in this study), *G.schlechtendaliana* starts to flower ca. 3–4 weeks earlier than *G.crassifolia*. Despite the slight overlap in their flowering periods, the temporal isolation could significantly reduce interspecific cross-pollination. In addition, the predominantly agamospermous breeding system of *G.crassifolia* helps maintain its reproductive isolation from *G.schlechtendaliana*. A similar reproductive isolation mechanism was proposed to explain the maintenance of integrity between sexually reproducing taxa and agamospermous taxa within the same genus (Catling and Brown 1983).

### ﻿Phylogenetic distinctness of *Goodyeracrassifolia*

**Figure 7.**
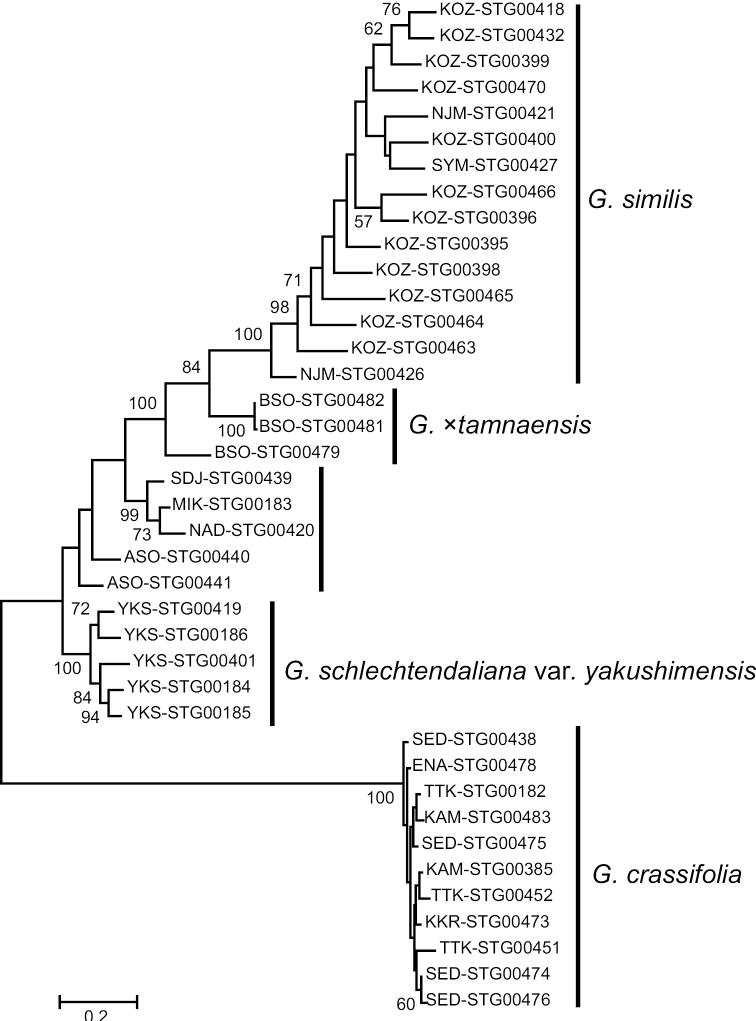
Phylogenetic tree of *Goodyeracrassifolia* and its closely related taxa reconstructed using MIG-seq data. Bootstrap values within species, and those less than 50%, are not shown. Branch length represents the average number of substitutions per site.

**Figure 8.**
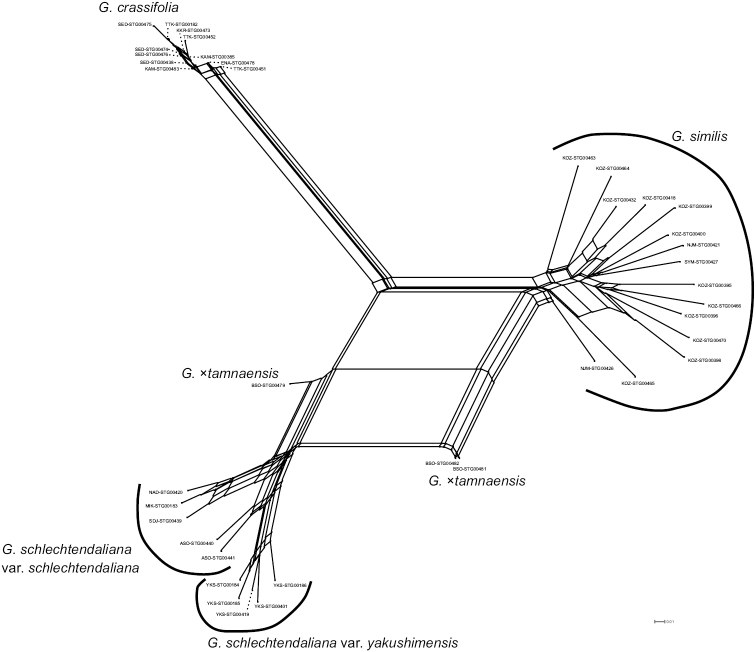
Neighbor-Net network for *Goodyeracrassifolia* and its closely related taxa, based on uncorrected P distances calculated from 4790 SNPs.

MIG-seq-based maximum likelihood phylogenetic tree generated in this study revealed that *G.crassifolia* forms a separate clade from *G.similis* and *G.schlechtendaliana* (100% bootstrap value: Fig. 7). *Goodyeraschlechtendaliana* was paraphyletic, while the monophyly of G.schlechtendalianavar.yakushimensis was supported (100% bootstrap value). Neighbor-Net phylogenetic analysis indicated that *G.crassifolia*, *G.schlechtendaliana*, and *G.similis* represent three distinct genetic clusters (Fig. 8). In the Neighbor-Net analysis, we show that the genetic diversity of *G.schlechtendaliana* as a whole, including G.schlechtendalianavar.yakushimensis, is comparable to that of *G.similis*. Therefore, G.schlechtendalianavar.yakushimensis is more likely to be an intraspecific variant of *G.schlechtendaliana* rather than an independent species. The interpretation is also based on the results of STRUCTURE analysis mentioned below, as well as on the relatively small morphological differences between var. schlechtendaliana and var. yakushimensis indicated by Suetsugu and Hayakawa (2019).

**Figure 9.**
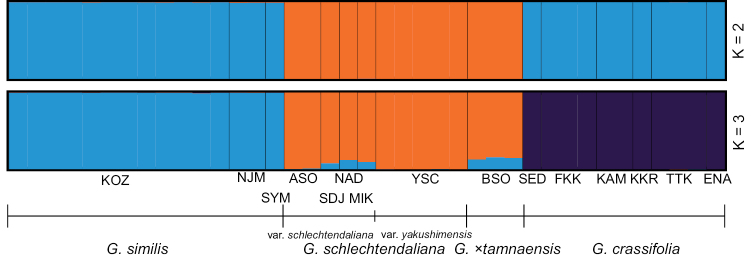
Population structure of *Goodyeracrassifolia* and its closely related taxa, inferred with STRUCTURE 2.3.4. Using *K* = 2 and *K* = 3 generated the largest and second-largest delta *K*, indicating that they were the most and second most optimal, respectively. Species and populations are separated by broad and narrow vertical black lines, respectively.

The STRUCTURE analysis at *K* = 2 (the largest delta *K* for our data) classified *G.crassifolia* and *G.schlechtendaliana* (including var. yakushimensis) into the same cluster, while at *K* = 3 (the second-largest delta *K*), *G.crassifolia*, *G.schlechtendaliana* (including var. yakushimensis), and *G.similis* formed three groups (Fig. 9). These findings, together with its multiple morphological differences from those observed in *G.schlechtendaliana* and *G.similis*, support the status of *G.crassifolia* as an independent species. Furthermore, genetic variation, which was high in the outcrossing *G.schlechtendaliana*, was low in the predominantly agamospermous *G.crassifolia*, both between and within populations. Similar patterns have been observed in other orchids, including *Nigritella* Rich., which includes both outcrossing and agamospermous species (Hedrén et al. 2018).

Molecular data obtained in this study provide further evidence that *G.crassifolia* has a different evolutionary origin from *G.×tamnaensis*. Both phylogenetic and population structure analyses showed that *G.×tamnaensis* has genetic components of both *G.schlechtendaliana* and *G.similis* (Figs 7–9). By contrast, although *G.crassifolia* was suspected as a natural hybrid of *G.schlechtendaliana* and *G.similis* (Takahashi 1985; Akiyama 2010), neither the phylogenetic analysis nor the population structure analyses support genetic admixture between *G.crassifolia* and any of its close congeners (Figs 7–9).

## ﻿Conclusion

The results obtained in this study confirm that *G.crassifolia* is distinct from *G.×tamnaensis*, refuting the hybrid origin hypothesis. Our rejection of the hybrid origin hypothesis is consistent with the karyological study of Sera (1990) concluding that the 2*n* = 60 plants (= *G.crassifolia*) are autopolyploids of the typical *G.schlechtendaliana*, given their similar resting-stage and mitotic-prophase chromosome morphology. Different chromosome number, agamospermous breeding, and early flowering possibly contributed to the premating isolation of *G.crassifolia* from its morphologically most similar species, the sympatric *G.schlechtendaliana*. Overall, the molecular phylogeny reconstructed from MIG-seq data together with morphological, cytological, and ecological analyses, support the separation of *G.crassifolia* as an independent species.

### ﻿Updated taxonomic treatment

#### ﻿ Goodyera crassifolia

Taxon classificationPlantaeAsparagalesOrchidaceae

H.-J.Suh, S.-W.Seo, S.-H.Oh & T.Yukawa

6ACD988C-E19A-5C3D-AFD1-D0D47A25C103

##### Type.

Korea. Jeollanam-do, Sinan-gun, Heuksando Island, 26 September 2016, *S.-H. Oh et al. 7155* (holotype: KB, isotypes: BH, TNS!, TUT).

Terrestrial herb, 20–37 cm tall. Rhizome pale green to brownish green, rooting at nodes. Roots fleshy, yellowish-brown, with minute root hairs. Stems erect, terete, 20–37 cm long, 3.4–7.5 mm in diam., pale green, glabrous. Leaves 5–15, widely spaced or somewhat clustered toward apex along the stem, 4.0–9.2 cm long; lamina ovate to lanceolate-ovate, 3.3–7.5 × 1.3–3.1 cm, length: width ratio 1.6–2.8, coriaceous, rounded at base, acute at apex, dorsally green with pale white reticulation or without any color decoration; petiole-like. Inflorescence a lax secund raceme, 6–14-flowered, with 2–4 sterile bracts; rachis 6.9–17.1 cm, internodes 17–24 mm long at inflorescence base; floral bracts lanceolate, 8–16 mm, pubescent, acuminate to acute at apex, pale green, shorter than the pedicellate ovary. Ovary and pedicel cylindric-fusiform, 11–20 mm, pale green, pubescent; hair on ovary and pedicel 0.3–0.5 mm, clavate. Flowers resupinate, weekly open. Sepals free, sub-similar, white tinged with pale yellow, pubescent on the outer surface, 1-veined; dorsal sepal narrowly elliptic-lanceolate, cymbiform, 10.1–12.8 × 3.3–4.4 mm, subacute at apex, forming a hood with petals; lateral sepals obliquely ovate-lanceolate, 9.7–12.5 × 3.2–4.8 mm, recurved at 2/3 of its entire length from the base, acute at apex, weekly spreading. Petals obliquely rhombic-oblanceolate to oblong-oblanceolate, 10.0–12.0 × 3.5–4.6 mm, hood recurved at apex, white tinged with pink or pale yellow, glabrous, 1-veined. Lip ovate-lanceolate, 9.5–11.5 × 2.7–4.0 mm; hypochile weekly concave-saccate, occasionally three-lobed, papillose inside; epichile ligulate, subacute at apex with 2 keels along the midrib. Column with lateral appendages; 5.8–7.3 mm long; stigma orbicular, slightly protruding; rostellar arms slender, occasionally three-lobed, sharp at apex; lateral appendage, rarely absent, usually 2 (–4), subulate or clavate, somewhat column-like, up to 6.0 mm long; anther ovate, 3.4–4.0 mm long; pollinia clavate, ca 4.0 mm; viscidium elliptic, ca. 2.0 mm long. Fruits cylindrical-fusiform, 13–22 mm long. Seeds fusiform, 0.8–1.1 mm long; embryo 1–3, ellipsoid, ca. 0.2 mm long.

##### Specimens examined.

**Japan. Kyushu District**—Miyazaki Pref.: Nishiusuki-gun, Gokase-cho, Kuraoka, 25 September 2013, *T. Minamitani s.n.* (AICH). Fukuoka Pref.: Kitakyushu-shi, Kokuraminami-ku, 11 September 2016, *K. Tanaka KS209* (KYO); Kitakyushu-shi, Kokuraminami-ku, 23 September 2018, *K. Tanaka STG00473* (KYO, herbarium sheet and spirit collection labelled as the same specimen); Tagawa-gun, Soeda-cho, Fukakura, 1 October 2016, *K. Tanaka STG00438* (KYO, spirit collection); Tagawa-gun, Soeda-cho, Fukakura, 24 September 2018, *Koji Tanaka STG00474* (KYO, herbarium sheet and spirit collection labelled as the same specimen); Kaho-cho, Mt. Kosyo, 4 May 1980, *T. Sera HIBG12487* (HIBG). **Shikoku District**—Ehime Pref.: Siyo-shi, Nomura-cho, Komatsu, 9 May 1981, *H. Yoshioka HIBG4684* (HIBG). Kochi Pref.: Agawa-gun, along Nano River, 21 July 1888, *s.n.* (TI); Takaoka-gun, Niyodo-mura, 13 September 1962, *G. Murata s.n.* (KYO); Bandamori, September 1889, *T. Makino s.n.* (MAK); Aki-gun, Kitagawa-mura, date unknown 1886, *S. Watanabe s.n.* (MAK); Kami-shi, Kahoku-cho, 17 September 2015, *H. Takeuchi & K. Suetsugu KS208* (KYO, spirit collection); Kami-shi, Kahoku-cho, 14 September 2016, *K. Suetsugu STG00385* (KYO, spirit collection); Kami-shi, Kahoku-cho, 28 September 2021, *H. Takeuchi G161-1* (KYO, herbarium sheet and spirit collection labeled as the same specimen); Muroto-shi, Sakihama-cho, 15 September 1974, *S. Takafuji s.n.* (KYO); Hata-gun, Hashigami-mura, 25 September 1914, *H. Yamaguchi s.n.* (TNS); Nyodogawa-cho, along Nakano River, 29 September 2020, *S. Hyodo KS767* (KYO, spirit collection). **Chugoku District**—Yamaguchi Pref.: Abu-gun, Akiragi-mura, 24 September 1919, *S. Nikai s.n.* (TNS). Hiroshima Pref.: Otake-shi, Kuritani-cho, Kokuribayashi, 9 September 2021, *K. Takeuchi et al. HIBG25924* (HIBG); Otake-shi, Kuritani-cho, Kokuribayashi, 9 September 2021 *K. Takeuchi et al. HIBG25925* (HIBG); Otake-shi, Kuritani-cho, Kokuribayashi, 9 September 2021, *K. Takeuchi et al. HIBG25926* (HIBG). Hyogo Pref.: Miki-shi, Fukui, 11 September 2021, *K. Umeki s.n.* (HYO). **Kinki District**—Nara Pref.: Totsukawa-mura. 26 September 2009, *K. Suetsugu KS207* (TNS); Yoshino-gun, Totsukawa-mura, 2 March 2017, *K. Suetsugu STG00182* (KYO); Yoshino-gun, Totsukawa-mura, 18 July 2018, *K. Suetsugu STG00451* (KYO). Wakayama Pref.: Nishimuro-gun, Kawazoe-mura, 23 September 1927, *N. Nakashima s.n.* (TI); Shingu-shi, Dorohaccho, 7 November 1950, *G. Nakai 5020* (KYO); Mt. Koya, 24–25 September 1955, *G. Murata s.n.* (KYO); Higashimuro-gun, Nachikatsuura-cho, September 1904, *K. Minakata s.n.* (MAK); Higashimuro-gun, Kogagawa-cho, 10 October 2021, *Y. Takada s.n.* (MAK); Arida-gun, Aridagawa-cho, Kusumoto, 29 September 2013, *A. Naitou 1592* (AICH). Mie Pref.: Kihoh-cho, Ainotani, 27 April 2009, *K. Suetsugu & T. Tonda KS206* (KYO); along Choshi River, 25 September 1955, *K. Iwatsuki s.n.* (KYO); Inabe-shi, Hokusei-cho, Betsumyo, 4 October 2013, *Y. Deguchi s.n.* (AICH). **Chubu District**—Gifu Pref.: Ena-shi, 16 September 2018, *K. Iwahori STG00478* (KYO, herbarium sheet and spirit collection labelled as the same specimen). Aichi Pref.: locality unknown, September 1897, collector unknown (KYO); Toyohashi-shi, Iwasaki-cho, Nagao, 28 September 2020, *Y. Kitada KS871* (KYO, spirit collection); Atsumi-gun, Atsumi-cho, Takaki, 24 September 2001, *M. Kobayashi 73668* (AICH); Higashikamo-gun, Asahi-cho, Yawata, 22 August 1992, *S. Serizawa 62497* (AICH); Toyota-shi, Sasabara-cho, 28 August 1991, *S. Serizawa 60088* (AICH); Toyota-shi, Tamomi-cho, Fujibora, 10 September 2007, *S. Serizawa 82210* (AICH); Nukata-gun, Kota-cho, Fukozu, 22 September 1995, *R. Kaneko 1275* (AICH); Hazu-gun, Kira-cho, Madarame, 11 March 1991, *H. Okada 28* (AICH); Seto-shi, Kawahira-cho, 12 September 1999, *T. Tsukamoto 2833* (AICH); Seto-shi, Sono-cho, 6 September 1999, *T. Tsukamoto 2828* (AICH); Seto-shi, Sono-cho, 25 September 2000, *T. Tsukamoto 2924* (AICH); Seto-shi, Anada-cho, 20 September 1992, *O. Hibino 856* (AICH); Seto-shi, Umagajo-cho, 26 September 1992, *T. Tsukamoto 397* (AICH); Seto-shi, Higashiyamaji-cho, 10 September 1998, *T. Tsukamoto 2701* (AICH); Seto-shi, Hirokute-cho, 21 September 1999, *S. Serizawa 76414* (AICH); Seto-shi, Uenoyama-cho, 20 September 2000, *T. Tsukamoto 2921* (AICH); Owariasahi-shi, Hirako-cho, 23 September 2013, *M. Muramathu 27088* (AICH); Komaki-shi, Oyama, 29 April 1997, *M. Kobayashi 60932* (AICH); Kasugai-shi, Hazama-cho, 18 September 2005, *K. Yamada 1256* (AICH); Nagoya-shi, Moriyama-ku, Togoku, 13 September 2008, *S. Serizawa 83258* (AICH); Nagoya-shi, Moriyama-ku, Kikko, 19 July 2017, *S. Serizawa 92748* (AICH). Shizuoka Pref.: Kosai-shi, Tame, 23 September 1995, *U. Naitou 5558* (AICH). **Kanto District**—Kanagawa Pref.: Sagamihara-shi, Midori-ku, 23 October 2010, *M. Nagai s.n.* (SCM). Tokyo Metropolis: Hachijo Island, 9 October 1974, *T. Nakaike 50067* (TNS).

##### Note.

Although Oh et al. (2022) noted that *G.crassifolia* is restricted to two offshore islands of the Korean peninsula and to a few locations in Japan, we have recognized many other new localities in Japan. Notably, all the *G.crassifolia* herbarium specimens (except the SCM specimen treated as G.×tamnaensis) have been annotated as *G.schlechtendaliana*. Therefore, *G.crassifolia* may have been misidentified as *G.schlechtendaliana* in the other areas. Extensive surveys during the flowering season are needed to elucidate the distribution of *G.crassifolia*.

## Supplementary Material

XML Treatment for Goodyera crassifolia

## Supplementary materials

Supplementary material 1 Newly collected materials used for morphological, cytological and MIG-seq analysis Data type excel file. https://binary.pensoft.net/file/765865This dataset is made available under the Open Database License (http://opendatacommons.org/licenses/odbl/1.0/). The Open Database License (ODbL) is a license agreement intended to allow users to freely share, modify, and use this Dataset while maintaining this same freedom for others, provided that the original source and author(s) are credited.Kenji Suetsugu, Shun K. Hirota, Narumi Nakato, Yoshihisa Suyama, Shunsuke Serizawa
